# Early real-life outcomes of dupilumab and mepolizumab in patients with uncontrolled primary diffuse type 2 chronic rhinosinusitis

**DOI:** 10.1007/s00405-025-09725-x

**Published:** 2025-10-18

**Authors:** Tommaso Saccardo, Giuseppe Roccuzzo, Nicola Tessari, Sonny Zampollo, Alessandro Fontana, Bruno Scarpa, Piero Nicolai, Giancarlo Ottaviano

**Affiliations:** 1 Department of Neurosciences, Otolaryngology Section DNS, University of Padova, Padova, Via Giustiniani 2, 35128 Italy; 2 Department of Statistical Sciences, Department of Mathematics Tullio Levi-Civita, University of Padova, Padova, Italy

**Keywords:** CRSwNP, Dupilumab, Mepolizumab, Real life, Comparison

## Abstract

**Purpose:**

Chronic rhinosinusitis with nasal polyps (CRSwNP) is a type 2 inflammatory disease causing significant morbidity. Biologics such as dupilumab (anti-IL-4Ra) and mepolizumab (anti-IL-5) are effective treatments; however, comparative real-world data remain limited. This study evaluates their effectiveness over the first six months in patients with uncontrolled primary diffuse Type 2 chronic rhinosinusitis.

**Methods:**

A retrospective analysis was conducted on 85 CRSwNP patients treated at Padova University Hospital (58 dupilumab, 27 mepolizumab). Patient-reported (SNOT-22, VAS scores) and clinician-reported (NPS, SSIT, PNIF) outcomes were assessed at baseline (T0), one month (T1), three months (T3), and six months (T6).

**Results:**

Dupilumab showed a rapid and significant improvement in all assessed PROMs and CROMs (*p* < 0.001), with a marked reduction in nasal polyp size and superior early olfactory recovery. By six months, 17% of dupilumab-treated patients achieved normosmia versus 7% in the mepolizumab group. Mepolizumab improved most outcomes except VAS-smell and significantly reduced blood eosinophil counts (*p* < 0.001), whereas dupilumab had no significant effect. According to EUFOREA 2023 criteria, 70% of dupilumab-treated patients had a moderate to excellent response versus 37% in the mepolizumab group.

**Conclusion:**

Both biologics are effective for CRSwNP, but their efficacy profiles differ. Dupilumab provides faster olfactory and nasal polyp improvements. Personalized biologic selection is essential, and further studies are needed to define long-term outcomes.

## Introduction

Chronic rhinosinusitis (CRS) is a chronic inflammatory disease affecting the nasal and paranasal sinus mucosa, defined by the persistence of symptoms for at least 12 weeks. Among its subtypes, CRS with nasal polyps (CRSwNP) represents approximately one third of cases and is primarily associated with type 2 inflammation [1, 2]. CRSwNP is characterized by debilitating symptoms such as nasal obstruction, hypo/anosmia, rhinorrhea, and facial discomfort, significantly impacting patients’ quality of life and healthcare resources [3]. Additionally, CRSwNP is often associated with comorbid conditions, including asthma, allergic rhinitis, and atopic dermatitis, which further exacerbate its clinical burden [4].

In recent years, biologic therapies have emerged as a revolutionary approach in the management of severe uncontrolled CRSwNP, consisting of monoclonal antibodies (mAb) targeting specific immune pathways involved in type 2 inflammation [5]. Among these therapies, dupilumab (anti-IL-4Ra mAb) and mepolizumab (anti-IL-5 mAb) have been demonstrated effective in reducing nasal polyp size, alleviating symptoms, and improving quality of life in randomized controlled trials [6, 7]. While these studies provide robust evidence of their individual efficacy, there remains a paucity of data on their comparative performance in real-world clinical settings [8].

This study aims to provide a direct, real-life comparison of the clinical effectiveness of dupilumab and mepolizumab in patients with severe CRSwNP treated at University Hospital of Padova (AOPD). By analyzing real-world outcomes during the first six months of treatment, this analysis seeks to complement existing clinical trial data, offering insights into the relative benefits of these therapies in routine clinical practice.

## Materials and methods

### Population

The present investigation consists of a retrospective analysis in a real-life setting involving the rhinological unit of AOPD. Consecutive patients starting biological therapy for severe uncontrolled CRSwNP between January 2022 and June 2024 for dupilumab, and March 2023 and June 2024 for mepolizumab were included in the present study. The study was conducted in accordance with the 1996 Helsinki Declaration and was approved by AOPD ethical committee (5304/AO/22 - AOP3240). Informed consent on personal data collection and use for research purposes was obtained from each subject before starting any treatment-related procedure.

Inclusion criteria were: (I) age ≥ 18 years; (II) diagnosis of severe CRSwNP, defined by a nasal polyp score (NPS) ≥ 5 and/or a Sinonasal Outcome Tests-22 (SNOT-22) ≥ 50 despite intranasal corticosteroids (INCS) use, receiving at least two cycles of systemic corticosteroid (SCS) in the previous 12 month year and/or having undergone one or more endoscopic sinus surgery (ESS) (according to the criteria of The Italian Medicines Agency) [9]; (III) administration of dupilumab 300 mg, one subcutaneous injection every two weeks or mepolizumab 100 mg, one sub-cutaneous injection every four weeks indicated for severe CRSwNP treatment as an add-on therapy to INCS as conventional treatment.

In all patients with a history of surgery, eligibility for biologic therapy required prior execution of at least a primary full-house functional endoscopic sinus surgery (FESS) or a complete revision ESS, performed at least 6 months prior to the initiation of biologic therapy, with no cases having undergone extended frontal sinusotomy [10]. In selected cases, biologic therapy was also considered in the presence of contraindications to general anesthesia or when repeated systemic corticosteroid use was not advisable due to comorbidities that limited the safe cumulative steroid dose.

The exclusion criteria were pregnancy, radio-chemotherapy for cancer in the 12 months before the start of the treatment, concomitant long-term oral corticosteroid therapy specifically indicated for other comorbidities, and Eosinophilic granulomatosis with polyangiitis (EGPA) diagnosis.

Bilateral diffuse CRS with predominance of type 2 inflammation in accordance with the criteria of the European Position Paper on Rhinosinusitis and Nasal Polyps (EPOS) 2020 [2] was evident in each patient. During the overlap prescription period (March 2023 – June 2024), due to a lack of clear biomarkers to guide the selection between biological therapies in CRSwNP treatment, the choice of dupilumab or mepolizumab was based on patients’ clinical history, baseline eosinophil blood count (EBC), comorbid asthma, patient counselling, and primary clinical burden. Generally, Mepolizumab was preferred in patients with a history of persistently elevated EBC (> 0.5 *10^9^/L and < 1.5 *10^9^/L) or significant eosinophilic comorbidities. In a minority of cases during patient counselling, mepolizumab was preferred because of its single monthly administration and its manageability. Dupilumab was preferred in patients with comorbid asthma with high FeNO or in patients mainly complaining of olfactory loss [11].

All patients included in the study were regularly using intra-nasal corticosteroids (INCS) before and throughout the whole study period. Asthmatic patients consistently used a combination of long-acting beta-agonists (LABAs) and inhaled corticosteroids (ICSs) without any interruptions in their treatment, if required.

Data were collected at the following timepoints: baseline (before starting the biological treatment) (T0), one month (T1), 3 months (T2), and 6 months (T3) after the first injection.

Demographic data, clinical and surgical history, allergological history (in particular inhalant allergies), total serum IgE, smoking habit and number of oral corticosteroids (OCS) courses in the previous 12 months were collected at T0. In addition, the Lund-Mackay score from a CT scan performed no more than 6 months before the initiation of biologic therapy was recorded [12].

At baseline and at each follow-up the following patients reported outcomes (PROMs) were systematically collected: asthma control test (ACT) score [13], SNOT-22 questionnaire [14], Nasal Congestion Score (NCS) test [15], visual analogue scale (VAS) for nasal obstruction (VAS-NO), olfactory impairment (VAS-smell), facial pain (VAS-facial pain) and sleep disturbance (VAS-sleep) [16]. Furthermore, the following clinicians reported outcomes (CROMs) were collected: peak nasal inspiratory flow (PNIF) (Clement Clark Inter- national, Mountain Ash, UK), to evaluate nasal patency [17]; Sniffin’ sticks identification sub-test (SSIT) (12 odors) (Burghart Messtechnik GmbH, Holm) [18], to categorize the smell impairment into anosmia, hyposmia and normosmia; nasal endoscopy using a 0◦ and or 30◦ rigid endoscope, during which NPS were collected [19]. Blood eosinophilia was evaluated at baseline and at each timepoint [20].

### Statistical analysis

Quantitative variables were presented as median and interquartile range [IQR], whereas categorical variables were expressed as number of observations and percentage (%).

Paired Wilcoxon test was also used to compare quantities between timepoints and treatment groups.

To assess whether baseline patient characteristics could predict therapeutic response based on EUFOREA 2023 criteria [15], we employed a proportional odds model. A hybrid stepwise backward selection procedure based on the Akaike Information Criterion (AIC) was applied to identify the most relevant predictors. The investigated variables included BMI, age at CRSwNP diagnosis, number of SCS cycles in the previous year, number of prior surgeries, months since the last surgery, presence of asthma, baseline eosinophilia, presence of inhalant allergies, and N-ERD (according to to EAACI criteria [21]).

For all tests p-values have been calculated, and 5% was considered as the critical level of significance. All the analyses have been performed in R (R Core Team, 2021) [22].

## Results

A cohort of 85 patients (64 males and 17 females, median age 56 years) undergoing biological treatment as add-on therapy were included in the present study. 58 were treated with dupilumab and 27 with mepolizumab. Patients’ demographics and both PROMS and CROMS at baseline (T0) are reported in Table 1. No dropouts occurred during the first 6 months of follow-up. All patients who initiated treatment with either dupilumab or mepolizumab completed the study period.

**Table 1:** Patients’ main clinical characteristics at baseline

Demographics		Sex	Age, yr median [IQR]	BMI, median [IQR]	Asthma, n (%)	NSAID intolerance, n (%)	N-ERD, n (%)	Allergy to inhalants, n (%)	IgE tot, median [IQR]	Smokers, n (%)	OCS*, median [IQR]	ESS, median [IQR]	
	**Dupilumab Group** n 58	13 Women (22%) 45 Men (78%)	55 [43–61]	25.8 [23.1–29.4]	35 (60.3%)	18 (31%)	16 (27.5%)	33 (56.8%)	183 [76–337]	22 (37.9%)	2 [2–3]	2 [1–3]	
	**Mepolizumab Group** n 27	8 Women (30%) 19 Men (70%)	57 [49.5–65]	25 [23.1–26.2]	21 (77.7%)	9 (33.3%)	7 (25.9%)	13 (48.1%)	185 [82–315]	4 (14.8%)	1 [0.5-2]	1 [1–2]	
	**p-value**	0.654	0.115	0.165	0.215	0.864	0.895	0.644	0.892	0.987	0.288	**0.042**	
**PROMs and CROMs**		**VAS-NO**,** median [IQR]**	**VAS-rhinorrea** ** median [IQR]**	**VAS-smell**,** median [IQR]**	**VAS-** **facial pain**, **median** **[IQR]**	**VAS-sleep**, **median** **[IQR]**	**SNOT-22**,** median [IQR]**	**PNIF**,** median [IQR]**	**SSIT**,** median [IQR]**	**NPS**,** median [IQR]**	**EBC**, **median [IQR]**	**ACT score**, **median [IQR]**	**Lund-Mackay score**, **median** **[IQR]**
	**Dupilumab Group** n 58	8 [7–9]	8 [5.3-9]	10 [8–10]	5 [2–8]	6 [2.8-8]	57.5 [42.8–69.8]	122.5 [87.5–170]	4 [3–6]	6 [5–6]	470 [293–755]	20 [16–24]	16 [13–19]
	**Mepolizumab Group** n 27	8 [7–9]	8 [6–9]	10 [9–10]	7 [1,5–8]	8 [3.5-9]	66 [36.5–76.2]	100 [65-152.5]	3 [2-4.5]	6 [6-6.25]	580 [460–890]	21 [15–22]	14 [13–18]
	**p-value**	0.881	0.634	0.502	0.556	0.211	0.664	0.092	0.115	0.089	0.170	0.669	0.755

Paired Wilcoxon test was used to compare measures between groups

*PROMs* patient’s reported outcomes measures; *CROMs* clinician’s reported outcomes measures; *IQR* inter-quartile range; *BMI* body mass index; *N-ERD* non-steroidal anti-inflammatory drugs exacerbated respiratory disease; *IgE tot* serum total IgE (kU/L); *OCS* oral corticosteroids; *ESS* endoscopic sinus surgery; *VAS-NO* Visual Analogue Scale for nasal obstruction; *VAS-smell* Visual Analogue Scale for smell impairment; *VAS-facial pain* visual analogue scale for facial pain; *VAS-sleep* Visual Analogue Scale for sleep disturbance; *SNOT-22* Sinonasal Outcome Test-22; *PNIF* Peak Nasal Inspiratory Flow (L/min); *SSIT* Sniffin’ Sticks Identification Test (12 odours); *NPS* Nasal Polyp Score; *EBC* eosinophils blood count (cells x10^6^/L).; *ACT* Asthma Control Test; *OCS short course per year

**Fig. 1:**
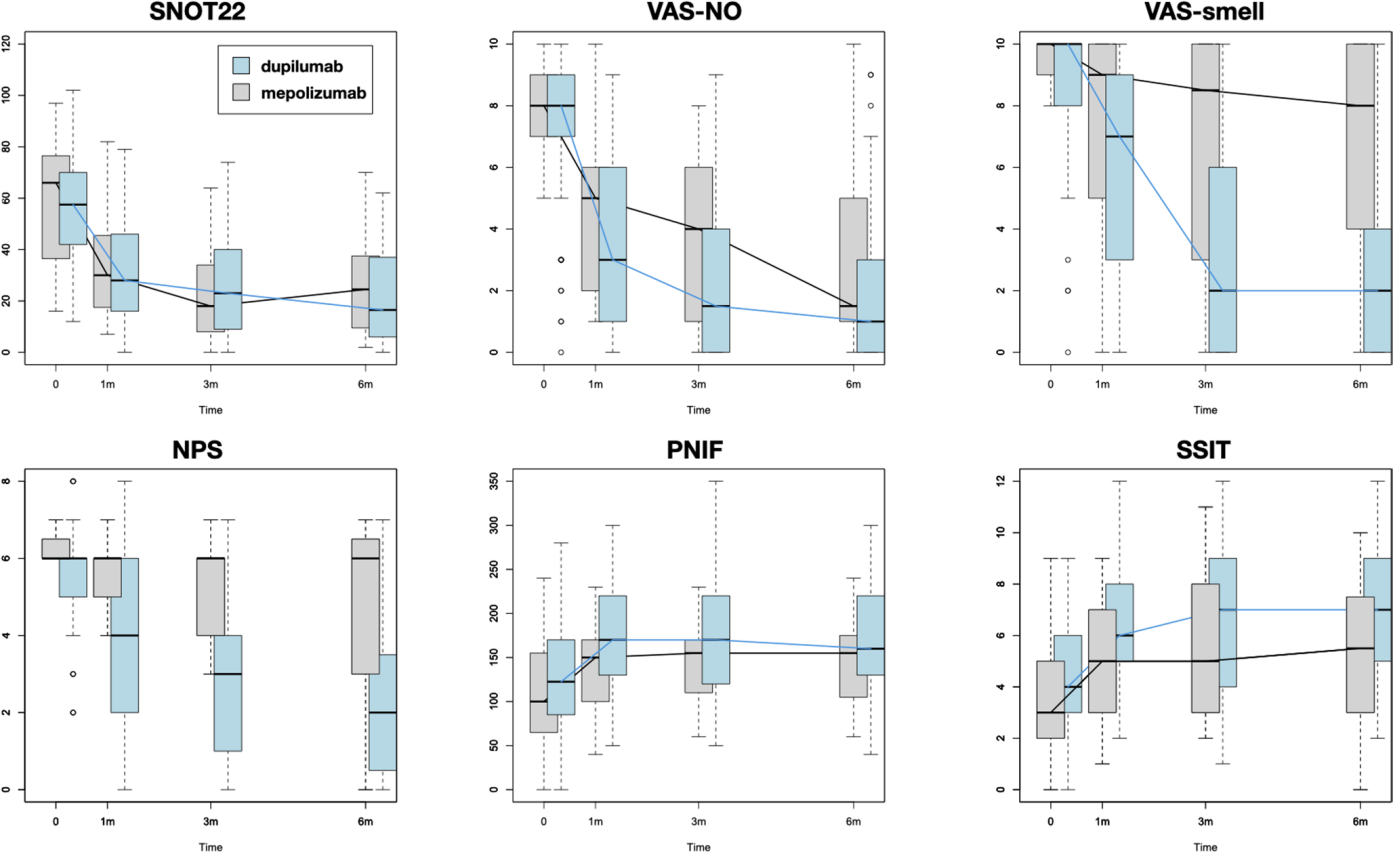
Main Patient Reported Outcome measures (PROMs) and Clinician’s reported outcomes measures (CROMs) changes during the study period SNOT-22: Sinonasal Outcome Test-22; VAS: Visual Analogue Scale; NO: Nasal Obstruction; NPS: nasal polyp score; PNIF: Peak Nasal Inspiratory Flow; SSIT: Sniffin’ Sticks Identification Test

In the dupilumab group a significant improvement was observed in all the evaluated nasal related PROMs (SNOT-22, NCS, VAS-NO, VAS-smell, VAS-rhinorrhea, VAS-sleep, and VAS-facial pain) and CROMs (NPS, SSIT, and PNIF) as early as the first month of treatment (*p* < 0.001) (Fig. 1). Similarly, a significant improvement (*p* < 0.05) in all the considered nasal related PROMs and CROMs was recorded in the mepolizumab group, with the exception of VAS-smell, which showed no statistically significant change (Fig. 1). At subsequent timepoints, the benefits observed in the first month were maintained in both treatment groups. Notably, in the dupilumab group, a further significant reduction in VAS-smell scores was observed between the first and third month (*p* = 0.006). Furthermore, in the mepolizumab group, a further significant reduction in VAS-rhinorrea scores was observed between the first and third month (*p* = 0. 017). No further statistically significant improvements were noticed in both groups at subsequent timepoints. No significant change was observed in ACT scores during the first 6 months of treatment in either the dupilumab or mepolizumab group (*p* > 0.05).

Olfactory outcomes demonstrated a difference in improvement between the two treatment groups. At baseline (T0), none of the patients in either group were normosmic. In particular, in the dupilumab group, 31% were hyposmic and 69% were anosmic; at T6, 17% of patients were normosmic, and 43% were classified as hyposmic. Conversely, in the mepolizumab group, 19% of patients were hyposmic at T0 and 81% were anosmic; by T6, 7% had achieved normosmia, while 44% remained hyposmic. When further analyzing patients’ olfactory function modification between T6 and T0 in both dupilumab and mepolizumab group, 53% and 34% patients respectively experienced an improvement in smell identification, leading to a step-up in their olfactory function categorization. 40% and 59% of patient belonging respectively to the dupilumab and mepolizumab group, retained their original categorization. Additionally, 7% of patients in both groups experienced a decline in smell sensitivity, resulting in a step-down in their olfactory function categorization.

During the same study period, EBC significantly decreased in the mepolizumab group both in the first month of treatment and between the first and the third month. No statistically significant changes were noted in EBC in the dupilumab group in the first 6 months of treatment.

When comparing the improvements between the two groups over time, the dupilumab-treated group showed significantly greater improvements in both VAS-smell and NPS scores compared to the mepolizumab-treated group. However, no statistically significant difference was observed neither in the SSIT nor in PNIF test results. Regarding EBC, mepolizumab significantly reduced circulating eosinophil counts, while dupilumab was associated with a slight, non-significant, increase. No significant differences emerged for the other considered variables between the two groups (Table 2).

**Table 2:** Differences of deltas of the main proms and CROMs between dupilumab and mepolizumab groups during the study period.

	T1vsT0		T3vsT0		T6vsT0	
	difference of Δ values between dupilumab and mepolizumab groups	*p*-value	difference of Δ values between dupilumab and mepolizumab groups	*p*-value	difference of Δ values between dupilumab and mepolizumab groups	*p*-value
VAS-NO	- 2	0.956	− 2.5	0.526	0	0.631
VAS-rhinorrhea	− 1	0.583	+ 1	0.075	0	0.569
VAS-facial pain	+ 1	0.586	+ 3	0.631	+ 2	0.652
VAS-smell	− 5	**0.001**	− 6.5	**0.002**	− 3	**0.005**
VAS-sleep	+ 1	0.804	+ 1	0.464	+ 1	0.468
SNOT22	+ 6.5	0.708	+ 13.5	0.291	+ 5	0.925
ACT	− 4	0.217	+ 2	0.460	+ 2	0.761
NCS	− 2	0.937	− 1	0.325	− 1	0.993
NPS	− 2	**0.008**	− 3	**< 0.001**	− 4	**< 0.001**
PNIF	− 2.5	0.744	− 7.5	0.753	− 12.5	0.544
SSIT	0	0.131	+ 1	0.604	0	0.606
EBC	+ 400	**< 0.001**	+ 593	**< 0.001**	+ 645	**< 0.001**

Δ: difference of median score between the two timepoints. Paired Wilcoxon test was used to compare differential measures between groups

*VAS* Visual Analogue Scale; *NO* Nasal Obstruction; *SNOT-22* Sinonasal Outcome Test-22; *ACT* Asthma Control Test; *NCS* Nasal Congestion Score; *NPS *Nasal Polyp Score; *PNIF* Peak Nasal Inspiratory Flow (L/min); *SSIT* Sniffin’ Sticks Identification Test; EBC: eosinophils blood count (cells x10^6^/L)

T0: baseline; T1: 1 month after the first administration; T3: 3 months after the first administration; T6: 6 months after the first administration

In multivariate analyses, conducted to assess the partial effects of various variables on clinical response given the effect of time, mepolizumab demonstrated a significant lower effect on NPS, PNIF, SSIT, and VAS-smell (*p* < 0.001) compared to dupilumab (Tables 3, 4, 5 and 6). However, no significant differences were observed for SNOT-22, NCS, VAS-NO, VAS-rhinorrhea, VAS-sleep, or VAS-facial pain.

**Table 3:** Multivariate analyses to identify the effects of the variables on NPS changes during the follow-up.

	Estimate	Std. Error	df	t value	Pr(>|t|)
(Intercept)	5.297	0.249	130.4869	21.270	*p* < 0.001
T1	−1.643	0.190	222.0336	−8.618	*p* < 0.001
T3	−2.250	0.199	224.3899	−11.273	*p* < 0.001
T6	−2.555	0.203	224.7938	−12.552	*p* < 0.001
Mepolizumab	**1.397**	**0.395**	**86.3422**	**3.530**	*p* < 0.001

**Table 4:** Multivariate analyses to identify the effects of the variables on SSIT changes during the follow-up.

	Estimate	Std. Error	df	t value	Pr(>|t|)
(Intercept)	4.521	0.304	162.3060	14.840	*p* < 0.001
T1	2.097	0.278	225.7246	7.541	*p* < 0.001
T3	2.095	0.287	228.3881	7.294	*p* < 0.001
T6	2.304	0.296	230.1600	7.787	*p* < 0.001
Mepolizumab	**−1.376**	**0.454**	**87.5931**	**−3.025**	*p* < 0.001

**Table 5:** Multivariate analyses to identify the effects of the variables on PNIF changes during the follow-up.

	Estimate	Std. Error	df	t value	Pr(>|t|)
(Intercept)	134.103	7.514	128.511	17.847	*p* < 0.001
T1	38.669	5.543	224.410	6.976	*p* < 0.001
T3	41.410	5.744	226.583	7.210	*p* < 0.001
T6	39.076	5.965	227.458	6.551	*p* < 0.001
Mepolizumab	**−26.661**	**11.950**	**85.029**	**−2.231**	*p* < 0.001

**Table 6:** Multivariate analyses to identify the effects of the variables on VAS-smell changes during the follow-up.

	Estimate	Std. Error	df	t value	Pr(>|t|)
(Intercept)	8.012	0.376	182.849	21.285	*p* < 0.001
T1	−3.326	0.382	219.257	−8.699	*p* < 0.001
T3	−4.611	0.388	221.071	−11.869	*p* < 0.001
T6	−4.941	0.407	225.010	−12.114	*p* < 0.001
Mepolizumab	**2.665**	**0.540**	**225.010**	**4.934**	*p* < 0.001

*NPS* nasal polyp score; Sniffin’ Sticks Identification Test; *ACT* Asthma Control Test; *PNIF* Peak Nasal Inspiratory Flow; T1: 1 month after the first administration; T3: 3 months after the first administration; T6: 6 months after the first administration

Regarding the safety profiles, a total of 22 patients (25.8% of the population) experienced minor/moderate adverse events during the study period: 16/58 patients (27.5%) during dupilumab and 6/27 (22%) during mepolizumab therapy. No major adverse events were evidenced in the population considered, and no patients discontinued treatment due to adverse events. In the dupilumab group, the most frequently reported adverse events were hypereosinophilia, injection-site dermatitis, and infectious events. Conversely, in the mepolizumab group, the most common adverse events included myalgia, epistaxis, and infectious events.

Considering the treatment response parameters after six months of therapy based on the 2023 EUFOREA criteria, in the dupilumab group, 70% of patients exhibited a moderate to excellent response, 27% had a poor response, and 3% showed no response. In contrast, in the mepolizumab group, the response rates were respectively 37%, 37% and 26%. During the six-month treatment period, no patient required systemic corticosteroids due to lack of symptom control or adverse events, nor did any patient require urgent surgical intervention for worsening disease. At the end of the observation period, continuation of biologic therapy was proposed to all patients, including those who had shown only limited improvement, in order to avoid prematurely discontinuing treatment in potential late responders [23].

None of the baseline variables investigated were found to be significant predictors of therapeutic response in the cumulative logit (proportional odds) model. The stepwise backward selection procedure based on the AIC did not retain any of these factors as independent predictors, indicating that none of them demonstrated a statistically significant association with treatment outcome.

## Discussion

Although indirect comparisons between biologics for CRSwNP are available in the literature [8, 24–27], there is a notable lack of robust head-to-head studies directly comparing their efficacy. At the time of writing, only one other study in the literature has evaluated the comparative effectiveness of dupilumab and mepolizumab [28]. This underscores the need for further research to establish clear guidance on the optimal biologic treatment for CRSwNP, particularly considering the nuanced differences in efficacy profiles observed in real-world settings. In this context, our study offers valuable insights into the real-world use of these biologics in managing CRSwNP over the first six months. While both biologics yielded significant improvements in PROMs and CROMs, notable differences in their efficacy profiles highlight the importance of personalized treatment selection. Specifically, dupilumab showed a greater reduction in nasal polyp size and more robust improvements in self-assessed olfactory function (VAS-smell). The latter result is of particular importance, especially when considering the difference in the mean number of ESS between the two study groups (Table 1). In fact, it has already been demonstrated by Alicandri-Ciufelli et al. that patients with prior extended and/or multiple surgeries had a decreased chance to improve their olfaction during the treatment with biologic therapy [29]. It is remarkable to note that, despite presenting with a significantly higher mean number of ESS, the dupilumab study group still showed a more consistent smell improvement, supporting its superiority in early smell perception recovery. In this regard 17% of patients achieved normosmia by six months in the dupilumab group compared to only 7% in the mepolizumab group, even though no statistically significant difference in SSIT improvement was found. Similar results were also reported in a recent study from De Santis et al. [28] in which 14.5% of the dupilumab group reached normosmia at 6 months, whilst none were nosmosmic in the mepolizumab group. These results are also consistent with previous findings of Oykhman et al. [30], who identified dupilumab as the most effective biologic for improving olfactory function in CRSwNP, outperforming both mepolizumab and omalizumab in an indirect comparison and meta-analysis of 20 randomized controlled trials.

The rapid improvement in olfactory outcomes observed in the dupilumab group is particularly noteworthy. Previous studies have shown that dupilumab can yield significant reductions in NPS and SNOT-22 scores as early as two weeks after initiation of treatment, with sustained benefits over time [31–33]. This rapid onset of action may provide critical QoL benefits for patients struggling with anosmia, a debilitating symptom commonly associated in Th2 CRS.

Interestingly, while dupilumab achieved significant clinical improvements in nasal and olfactory symptoms within the first few months of therapy, the improvement in olfactory function appear to gradually plateau after six months according to current literature [31, 34]. This pattern suggests an early and robust response in smell perception with dupilumab, followed by a stabilization phase beyond the initial six months of treatment. In contrast, recent real-life studies [35] suggest that the olfactory outcomes with mepolizumab may follow a different trajectory, with improvements extending beyond the six-month mark. This delayed but progressive improvement warrants further investigation to determine whether prolonged treatment with mepolizumab could yield a more sustained recovery in olfactory function.

The reduction in nasal polyp size was more pronounced in the dupilumab group compared to mepolizumab, which may explain the greater improvement in PNIF observed with dupilumab. Although no significant differences emerged at the univariate analysis, mepolizumab demonstrated a significant lower effect on PNIF improvement in the multivariate analysis compared to dupilumab. This suggests that the smaller reduction in nasal polyp size associated with mepolizumab may have limited its impact on nasal airflow dynamics. These findings are consistent with those of Papacharalampous et al. [24], who reported a 35% reduction in NPS with dupilumab, compared to 15% with mepolizumab. Nevertheless, despite showing some less pronounced effects, mepolizumab treatment still remains effective in reducing most PROMs and CROMs, making it a viable option, particularly for patients with concurrent eosinophilic manifestations. In fact, mepolizumab was found to significantly reduce circulating eosinophil levels compared to dupilumab, which may represent a key advantage since hypereosinophilia was one of the most frequently reported adverse events in the dupilumab group. However, while blood eosinophil levels are effectively reduced by mepolizumab, the extent to which mucosal eosinophil burden influences therapeutic outcomes remains uncertain. Considering the stability of mucosal eosinophils as a biopsy marker [36], it would be of particular interest to determine the baseline threshold of eosinophilic infiltration [37] that correlates with the greatest therapeutic response in the mepolizumab group [38]. This remains an open question and could serve as a key focus for future studies.

Beyond these observations on nasal outcomes, the safety profile of the two biologics was also evaluated in our cohort, allowing for a comprehensive overview of their short-term performance.

During the six-month follow-up, mild to moderate adverse events occurred in 25.8% of patients in our cohort. No patient discontinued biologic therapy despite this relatively high incidence, indicating a good short-term safety profile. This contrasts with both real-world evidence and clinical trial data, where discontinuation due to adverse events has been documented at varying rates. In the dupilumab phase III SINUS-24 and SINUS-52 studies, adverse events led to discontinuation in 2% of patients, compared with 5% in the placebo group [6]. At one year, a German real-world cohort of 81 patients reported eight discontinuations (38%), only one of which was associated with a severe adverse event [39]. The DUPIREAL study, on the other hand, reported a higher discontinuation rate (37.1%), with six cases attributed to severe side effects such as eosinophilic pneumonia and arthralgia [31]. Similarly, Ciofalo et al. observed a 5.9% rate of serious adverse events after six months, with two cases resulting in treatment discontinuation [40].

Our findings for mepolizumab are consistent with those of Gevaert et al., who reported that over 48 weeks, 53% of patients experienced at least one adverse event, none of which required discontinuation [41]. In a prospective Italian study of 30 patients, only one discontinued therapy, and 24% experienced minor injection-site reactions [35]. According to data from the LIBERTY NP trials, treatment-related adverse events occurred in 15% of mepolizumab patients compared with 9% in the placebo group [6].

Therapeutic effectiveness was further assessed using the standardized EUFOREA 2023 response criteria. The dupilumab group exhibited a higher response rate according to the EUFOREA 2023 criteria compared to mepolizumab (70% vs. 37% in moderate to excellent response and 3% vs. 26% in no response), suggesting a more rapid and pronounced therapeutic effect within the study period. However, it is possible that mepolizumab exerts a slower therapeutic effect, with its full benefits potentially extending beyond the first sixth month of treatment [35]. This delayed response could be attributed to differences in mechanisms of action, particularly regarding the time required for structural and inflammatory modifications in the sino-nasal mucosa. Future studies should focus on assessing the comparative long-term efficacy, aiming to determine whether mepolizumab therapeutic response continues to improve beyond the initial treatment phase.

Our findings emphasize the importance of considering patient preferences and symptom priorities when selecting biologics. Dupilumab’s marked improvement in olfactory function may be particularly advantageous for patients prioritizing the restoration of smell, while mepolizumab’s efficacy in reducing eosinophil counts may make it better suited for those with particularly high EBC at baseline. Further characterization of the patient population is needed to identify which individuals may benefit more from one therapy over the other. Unfortunately, in our cohort, no baseline characteristic emerged as a clear determinant to guide this therapeutic choice.

Lastly, considering the potential impact of biologic therapy on asthma comorbidities, it is worth noting that ACT scores did not show significant variation over the six-month follow-up period. This finding is not unexpected, as baseline scores already reflected good asthma control in the majority of patients, and asthma was not the primary driver for biologic prescription in this cohort. These factors likely contributed to the absence of a substantial change in asthma-related outcomes during the study.

## Limitations

The present study shows some limitations. The monocentric, retrospective design, the relatively small sample size, and the duration of the study period may limit the generalizability of the findings, particularly in the mepolizumab group. Potential confounding factors should also be acknowledged. Differences in baseline characteristics, such the extent and number of previous ESS procedures or the prevalence of comorbid asthma, may have influenced treatment outcomes. Moreover, other unmeasured patient-specific factors, such as variations in disease duration, inflammatory burden, or adherence to prescribed therapies, could have contributed to the observed differences between groups.

Future research should focus on prospective, multicenter studies comparing biologics in larger, more diverse cohorts. Additionally, further exploration of biomarkers and their correlation with clinical outcomes may provide valuable insights into treatment mechanisms and optimization. Comparative studies investigating long-term outcomes, including the need for surgical intervention, will also help refine treatment strategies for type 2 CRS.

## Conclusions

In conclusion, both dupilumab and mepolizumab are effective add-on therapies for CRSwNP, but their efficacy profiles differ in clinically meaningful ways. Dupilumab offers greater benefits in olfactory function and nasal polyp reduction, making it a preferred option for patients with significant anosmia or refractory disease. On the other hand, mepolizumab’s targeted suppression of eosinophilic inflammation may be advantageous for patients with higher EBC at baseline. These findings should be interpreted considering the retrospective, single-center design, the small sample size, and potential baseline differences between groups, but they nonetheless support a personalized approach to biologic selection. These findings reinforce the importance of tailoring biologic therapy to the specific needs and comorbidities of individual patients.

## Data Availability

The datasets generated and analysed during the current study are available on reasonable request.

## References

[1] Dietz de Loos D, Lourijsen ES, Wildeman MAM et al (2019) Prevalence of chronic rhinosinusitis in the general population based on sinus radiology and symptomatology. J Allergy Clin Immunol 143:1207–1214. 10.1016/j.jaci.2018.12.986 30578880 10.1016/j.jaci.2018.12.986

[2] Fokkens WJ, Lund VJ, Hopkins C (2020) European position paper on rhinosinusitis and nasal polyps 2020. Rhinology 0:1–464. 10.4193/Rhin20.600 10.4193/Rhin20.600 32077450

[3] Ottaviano G, Saccardo T, Roccuzzo G et al (2022) Effectiveness of dupilumab in the treatment of patients with uncontrolled severe crswnp: a real-life observational study in Naïve and Post-Surgical patients. J Pers Med. 10.3390/jpm12091526 36143311 10.3390/jpm12091526 PMC9502990

[4] Khan AH, Gouia I, Kamat S et al (2023) Prevalence and severity distribution of type 2 inflammation-related comorbidities among patients with asthma, chronic rhinosinusitis with nasal polyps, and atopic dermatitis. Lung 201:57–63. 10.1007/s00408-023-00603-z 36808551 10.1007/s00408-023-00603-z PMC9968259

[5] Ottaviano G, De Corso E, Saccardo T et al (2023) Effectiveness of dupilumab in the treatment of adult and older adult patients with severe, uncontrolled CRSwNP. J Pers Med. 10.3390/jpm13081241 37623491 10.3390/jpm13081241 PMC10456067

[6] Bachert C, Han JK, Desrosiers M et al (2019) Efficacy and safety of dupilumab in patients with severe chronic rhinosinusitis with nasal polyps (LIBERTY NP SINUS-24 and LIBERTY NP SINUS-52): results from two multicentre, randomised, double-blind, placebo-controlled, parallel-group phase 3 trials. Lancet 394:1638–1650. 10.1016/S0140-6736(19)31881-1 31543428 10.1016/S0140-6736(19)31881-1

[7] Han JK, Bachert C, Fokkens W et al (2021) Mepolizumab for chronic rhinosinusitis with nasal polyps (SYNAPSE): a randomised, double-blind, placebo-controlled, phase 3 trial. Lancet Respir Med 9:1141–1153. 10.1016/S2213-2600(21)00097-7 33872587 10.1016/S2213-2600(21)00097-7

[8] Hopkins C, Han JK, Fokkens W (2024) Dupilumab versus mepolizumab for chronic rhinosinusitis with nasal polyposis: an indirect treatment comparison. The Journal of Allergy and Clinical Immunology: In Practice 12:3393-3401e15. 10.1016/j.jaip.2024.09.015 39326524 10.1016/j.jaip.2024.09.015

[9] (2023) Gazzetta ufficiale Della repubblica Italiana. 2023. Decreto Del Presidente Del consiglio dei ministri 24 febbraio 2023. Gazz Ufficiale Serie Generale 51

[10] Saccardo T, Nicolas V, Chebib E et al (2024) Enlarged frontal sinusotomy and chronic rhinosinusitis with nasal polyps: an effective strategy to control the disease. Ann Otol, Rhinol Laryngol. 10.1177/00034894241298749 10.1177/00034894241298749 39529206

[11] Ottaviano G, De Corso E, Cantone E et al (2023) Measuring nasal patency and the sense of smell in CRSwNP patients treated with Dupilumab. J Pers Med. 10.3390/jpm13020234 36836468 10.3390/jpm13020234 PMC9962970

[12] Lund VJ, Mackay IS (1993) Staging in rhinosinusitus. Rhinology 31:183–184 8140385

[13] Ottaviano G, Roccuzzo G, Saccardo T et al (2025) Mepolizumab in severe uncontrolled crswnp: a real-life multicentre study in Northeast Italy. Rhinology 0:0–0. 10.4193/Rhin24.420 10.4193/Rhin24.420 40249916

[14] Mozzanica F, Preti A, Gera R et al (2017) Cross-cultural adaptation and validation of the SNOT-22 into Italian. Eur Arch Otorhinolaryngol 274:887–895. 10.1007/s00405-016-4313-x 27677485 10.1007/s00405-016-4313-x

[15] Fokkens WJ, Viskens A-S, Backer V et al (2023) EPOS/EUFOREA update on indication and evaluation of biologics in chronic rhinosinusitis with nasal polyps 2023. Rhinology J 0. 10.4193/Rhin22.489 10.4193/Rhin22.489 36999780

[16] Sedaghat AR, Campbell RG, Douglas RG et al (2024) Outcome measures for chronic rhinosinusitis with nasal polyps. Rhinology 0:0–0. 10.4193/Rhin24.090 10.4193/Rhin24.090 38829175

[17] Ottaviano G, Pendolino AL, Nardello E et al (2019) Peak nasal inspiratory flow measurement and visual analogue scale in a large adult population. Clin Otolaryngol 44:541–548. 10.1111/coa.13329 30887705 10.1111/coa.13329

[18] Whitcroft KL, Altundag A, Balungwe P et al (2023) Position paper on olfactory dysfunction: 2023. Rhinology 61:1–108. 10.4193/Rhin22.483 37454287 10.4193/Rhin22.483

[19] Gevaert P, De Craemer J, Bachert C et al (2023) European academy of allergy and clinical immunology position paper on endoscopic scoring of nasal polyposis. Allergy 78:912–922. 10.1111/all.15650 36661567 10.1111/all.15650

[20] Caminati M, Micheletto C, Norelli F et al (2024) Safety of dupilumab in T2 airways conditions: focus on eosinophilia across trials and real-life evidence. Expert Opin Biol Ther 24:15–23. 10.1080/14712598.2024.2304556 38197326 10.1080/14712598.2024.2304556

[21] Kowalski ML, Agache I, Bavbek S, et al (2019) Diagnosis and management of < scp > NSAID</scp>-Exacerbated Respiratory Disease (N‐ <scp > ERD</scp>)—a < scp > EAACI</scp > position paper. Allergy 74:28–39. 10.1111/all.13599 10.1111/all.13599 30216468

[22] R Core Team (2020) R: A Language and. Environment for Statistical Computing

[23] Pham DD, Lee J-H, Kwon H-S et al (2024) Predictors of early and late lung function improvement in severe eosinophilic asthma on Type2-biologics in the PRISM study. Lung 202:41–51. 10.1007/s00408-024-00670-w 38252134 10.1007/s00408-024-00670-w

[24] Papacharalampous GX, Constantinidis J, Fotiadis G et al (2024) Chronic rhinosinusitis with nasal polyps (CRSwNP) treated with omalizumab, dupilumab, or mepolizumab: a systematic review of the current knowledge towards an attempt to compare agents’ efficacy. Int Forum Allergy Rhinol 14:96–109. 10.1002/alr.23234 37394893 10.1002/alr.23234

[25] Tănase MI, Tanase M, Cosgarea M, et al (2025) Biologic Treatments for Chronic Rhinosinusitis With Nasal Polyps (CRSwNP): A Comparative Review of Efficiency and Risks. Cureus. 10.7759/cureus.77804 10.7759/cureus.77804 PMC11751697 39844880

[26] Barroso B, Valverde-Monge M, Betancor D et al (2023) Improvement in smell using monoclonal antibodies among patients with chronic rhinosinusitis with nasal polyps: a systematic review. J Investig Allergol Clin Immunol 33:419–430. 10.18176/jiaci.0939 37669083 10.18176/jiaci.0939

[27] Wu Q, Zhang Y, Kong W et al (2022) Which is the best biologic for nasal polyps: Dupilumab, Omalizumab, or mepolizumab? A network meta-analysis. Int Arch Allergy Immunol 183:279–288. 10.1159/000519228 34607329 10.1159/000519228

[28] De Santis S, Galassi S, Cambi J (2024) Clinical effects and response time of biological drugs in chronic rhinosinusitis with nasal polyps patients: real-life experience. Laryngoscope. 10.1002/lary.31948 39651676 10.1002/lary.31948

[29] Alicandri-Ciufelli M, Marchioni D, Pipolo C et al (2024) Influence of prior endoscopic sinus surgery extent on dupilumab effectiveness in < scp > CRSwNP Patients. Laryngoscope 134:1556–1563. 10.1002/lary.30983 37632705 10.1002/lary.30983

[30] Oykhman P, Paramo FA, Bousquet J et al (2022) Comparative efficacy and safety of monoclonal antibodies and aspirin desensitization for chronic rhinosinusitis with nasal polyposis: a systematic review and network meta-analysis. J Allergy Clin Immunol 149:1286–1295. 10.1016/j.jaci.2021.09.009 34543652 10.1016/j.jaci.2021.09.009

[31] De Corso E, Pasquini E, Trimarchi M et al (2023) Dupilumab in the treatment of severe uncontrolled chronic rhinosinusitis with nasal polyps (< scp > CRSwNP): A multicentric observational phase IV real-life study (< scp > DUPIREAL). Allergy 78:2669–2683. 10.1111/all.15772 37203259 10.1111/all.15772

[32] Hellings PW, Peters AT, Chaker AM et al (2022) Rapid and sustained effects of dupilumab in severe chronic rhinosinusitis with nasal polyps. Int Forum Allergy Rhinol 12:958–962. 10.1002/alr.22944 34911163 10.1002/alr.22944 PMC9306796

[33] Ottaviano G, Roccuzzo G, Lora L et al (2024) The impact of dupilumab on work productivity and emotional health in crswnp: a multicentric study in Northeast Italy. J Pers Med 14:468. 10.3390/jpm14050468 38793051 10.3390/jpm14050468 PMC11121907

[34] Huber P, Förster-Ruhrmann U, Olze H et al (2024) Real‐world data show sustained therapeutic effects of dupilumab in chronic rhinosinusitis with nasal polyps (< scp > CRSwNP) over 3 years. Allergy 79:3108–3117. 10.1111/all.16263 39109388 10.1111/all.16263

[35] Galletti C, Sireci F, Stilo G et al (2025) Mepolizumab in chronic rhinosinusitis with nasal polyps: real life data in a multicentric Sicilian experience. Am J Otolaryngol 46:104597. 10.1016/j.amjoto.2024.104597 39826192 10.1016/j.amjoto.2024.104597

[36] Sombutpiboonphon K, Snidvongs K, Lawpoolsri S et al (2024) Fluctuation of tissue eosinophils in chronic rhinosinusitis with nasal polyp. Int Forum Allergy Rhinol. 10.1002/alr.23494 39679984 10.1002/alr.23494

[37] Walter S, Ho J, Alvarado R et al (2022) Mepolizumab decreases tissue eosinophils while increasing type-2 cytokines in eosinophilic chronic rhinosinusitis. Clin Exp Allergy 52:1403–1413. 10.1111/cea.14152 35475305 10.1111/cea.14152

[38] Png LH, Kalish L, Campbell RG et al (2024) Predictors of persistent disease in biologic treated type 2 diffuse/eosinophilic chronic rhinosinusitis undergoing surgery. Int Forum Allergy Rhinol 14:909–918. 10.1002/alr.23282 37805956 10.1002/alr.23282

[39] Albrecht T, Sailer MM, Capitani F et al (2023) Real-world evidence for the effectiveness and safety of dupilumab in patients with CRSwNP after 1 year of therapy. World Allergy Organ J 16:100780. 10.1016/j.waojou.2023.100780 37234094 10.1016/j.waojou.2023.100780 PMC10206757

[40] Orlando P, Licci G, Illiano G et al (2025) Sustained efficacy and low rate of adverse events of dupilumab in Type-2 < scp > CRSwNP Over 48 months. Laryngoscope. 10.1002/lary.32205 40265743 10.1002/lary.32205

[41] Gevaert P, Van Bruaene N, Cattaert T et al (2011) Mepolizumab, a humanized anti–IL-5 mAb, as a treatment option for severe nasal polyposis. J Allergy Clin Immunol 128(5):989-995e8. 10.1016/j.jaci.2011.07.056 21958585 10.1016/j.jaci.2011.07.056

